# Structure Property–Application Relationships of Spinel Ferrite Nanoparticles: From Synthesis to Functional Systems

**DOI:** 10.3390/ijms27052096

**Published:** 2026-02-24

**Authors:** Mukhametkali Mataev, Altynai Madiyarova, Moldir Abdraimova, Zhanar Tursyn, Krishnamoorthy Ramachandran

**Affiliations:** 1 Department of Chemistry, Faculty of Natural Sciences, Kazakh National Women’s Teacher Training University, Almaty 050000, Kazakhstan; mataev.m@qyzpu.edu.kz (M.M.); tursyn.zh@qyzpu.edu.kz (Z.T.); 2 Department of Physics, SRM Institute of Science and Technology, Vadapalani Campus, Chennai 600026, India; ramachak1@srmist.edu.in

**Keywords:** spinel ferrites, ferrite nanoparticles, synthesis methods, cation distribution, magnetic properties, dielectric properties, structural characterization, catalysis, water treatment, biomedical applications

## Abstract

This review article provides a systematic analysis of synthesis methods, structural characteristics, and functional properties of spinel-structured ferrite nanoparticles (MFe_2_O_4_). The physicochemical principles, advantages, and limitations of various synthesis techniques—including co-precipitation, combustion, sol–gel, thermal decomposition, hydrothermal, solvothermal, microwave-assisted, sonochemical, electrochemical, and solid-state reaction methods—are comparatively discussed. The influence of synthesis parameters on crystal structure, morphology, and cation distribution between tetrahedral and octahedral sites, as well as on magnetic, dielectric, and optical properties, is critically analyzed. Furthermore, the capabilities of characterization techniques such as X-ray diffraction (XRD), scanning electron microscopy with energy-dispersive spectroscopy (SEM/EDS), Fourier-transform infrared spectroscopy (FTIR), FT-Raman spectroscopy, dielectric measurements, and magnetic measurements for investigating spinel ferrites are comprehensively summarized. Finally, the high potential of spinel ferrite nanoparticles for applications in electronics, microwave devices, water treatment, catalysis, sensors, and biomedical fields is highlighted.

## 1. Introduction

Research in nanotechnology has rapidly emerged as a leading field in modern physics and has attracted significant interest from the scientific community [1]. Multifunctional materials, in particular, have been extensively investigated due to the integration of multiple efficient properties within a single intelligent compound [2,3,4].

Based on their crystalline structures, nanomaterials can be classified into four major groups: spinel ferrites, garnet ferrites, hexaferrites, and orthoferrites [5]. Nanoferrites are magnetic materials composed of metal oxides and are widely employed across various technological fields [6]. Among them, spinel ferrites have gained considerable attention as prominent nanomaterials owing to their unique physicochemical properties and broad range of applications, including biomedical fields [7], water treatment, and industrial electronic devices [8,9].

**Figure 1:**
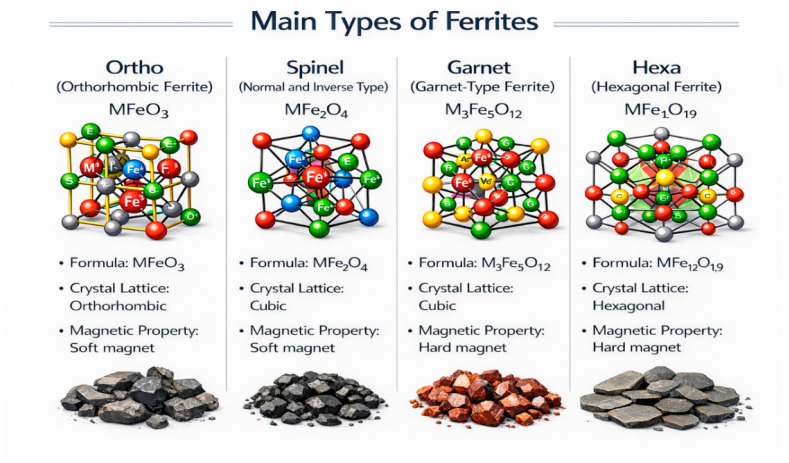
The four classes of ferrites: garnet-type ferrites, orthoferrites, hexagonal ferrites, and spinel ferrites.

Ferrites are characterized by high electrical resistivity and magnetic permeability, along with low dielectric losses and eddy current losses; therefore, they are extensively used in numerous electronic devices, computers, and microwave components [10]. All ferrites exhibit ferrimagnetic or ferromagnetic behavior and can be further categorized into four additional classes based on their crystal structures: garnets, orthoferrites, hexagonal ferrites, and spinel ferrites, as illustrated in Figure 1.

Ferrites are ferrimagnetic materials characterized by the spatial arrangement of oxygen anions and metal cations in well-defined geometric configurations within a crystalline lattice. Owing to their excellent magnetic, structural, and catalytic properties, ferrites are widely utilized in various applications. The intrinsic properties of ferrites—such as their structural features, electrical behavior, and magnetic response—are strongly influenced by the synthesis method, chemical composition, and cation distribution. Ferrites can be classified into four main types: spinel ferrites, hexaferrites, garnets, and orthoferrites [11]. Among these, spinel ferrites are considered one of the most promising materials due to their superior structural, magnetic, and catalytic properties.

Spinel ferrites, with the general chemical formula MFe_2_O_4_ (where M represents divalent ions such as Fe^2+^, Zn^2+^, Ni^2+^, and Co^2+^), have been recognized as highly efficient metal-based catalysts. Their effectiveness arises from several intrinsic characteristics that make them particularly suitable for catalytic applications [12], including high catalytic activity, facile synthesis, and excellent recyclability. The catalytic performance is primarily governed by the cation distribution, which allows precise tuning of their catalytic properties. The use of spinel ferrites dates back more than three millennia, with magnetite (Fe_3_O_4_) being one of the earliest known magnetic materials [13].

Spinel ferrite nanoparticles (NPs) are of great scientific interest because they enable direct investigation of the relationship between crystal chemistry and magnetic properties. By varying the composition and concentration of divalent cations, several key properties of ferrite nanoparticles can be tailored, making them suitable for applications in data storage [14], environmental remediation [15], energy conversion and storage [16], biomedicine [17], and medical diagnostics [18]. The wide range of potential applications of spinel ferrite nanoparticles has driven the development of numerous synthetic approaches for their production. The magnetic properties of spinel ferrite nanoparticles are among their most intriguing features, as they often differ significantly from those of their bulk counterparts [19,20]. In general, the magnetic behavior of nanoparticles is governed by size effects and surface phenomena. Size effects give rise to two important phenomena: superparamagnetism and the formation of single magnetic domains. Single-domain structures occur in nanoparticles with diameters below a critical size, where the formation of domain walls becomes energetically unfavorable, resulting in high magnetic anisotropy and a large magnetic moment per unit volume [21]. Magnetic anisotropy represents the energy barrier that stabilizes magnetic moments along a preferred direction. As particle size decreases further, thermal energy can overcome this barrier, leading to random fluctuations of magnetic moments—a phenomenon known as superparamagnetism [22].

Over the past decade, metal oxides and ferrite-based nanomaterials have been intensively investigated for energy-related applications due to their availability, low cost, stability, environmental friendliness, and ease of synthesis [23,24,25,26,27,28]. This is particularly true for first-row transition metal spinel ferrites (MFe_2_O_4_), which exhibit attractive properties such as high specific capacity, narrow energy bandgap, and rich redox chemistry, in addition to intrinsic magnetic characteristics including high saturation magnetization, low coercivity, and high Curie temperature [29,30]. Several recent review articles have examined the use of spinel ferrites, ferrites, and transition metal oxides in photoelectrochemical cells—such as water-splitting systems and dye-sensitized solar cells—highlighting the factors that limit photoelectrode performance and discussing strategies to overcome these challenges in detail [29,31,32,33,34,35,36,37].

**Figure 2:**
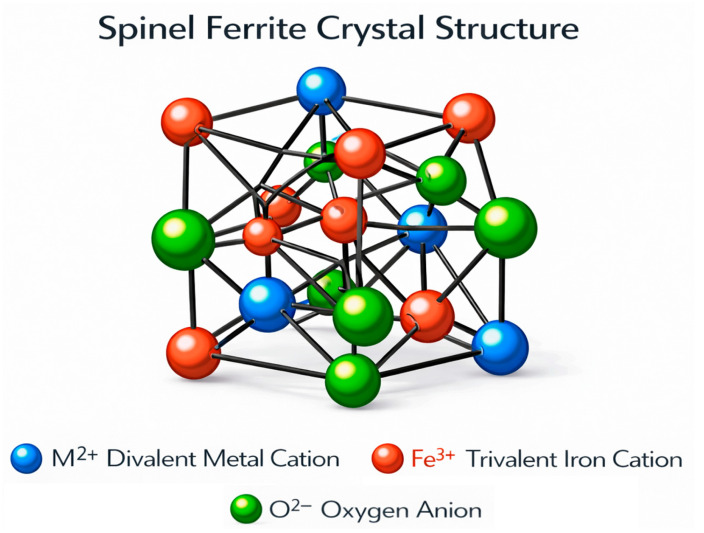
Schematic representation of the crystal structure of spinel ferrites.

Figure 2 illustrates the cubic close-packed array of oxygen atoms that constitutes the crystal structure of spinel ferrites. The chemical formula of spinel ferrites is MeFe_2_O_4_, where Me^2+^ and Fe^3+^ ions occupy two distinct crystallographic sites: tetrahedral (A-sites) and octahedral (B-sites). The coordination of oxygen atoms differs for each site. Specifically, each formula unit contains eight tetrahedral sites and sixteen octahedral sites. In a normal spinel configuration, Me^2+^ ions occupy the A-sites, while Fe^3+^ ions reside at the B-sites. In contrast, in an inverse spinel configuration, Fe^3+^ ions fully occupy the A-sites, whereas the B-sites are shared between Fe^3+^ and Me^2+^ cations. Many spinel ferrites exhibit partial inversion, in which both Me^2+^ and Fe^3+^ ions are distributed between the A- and B-sites. These materials typically display ferrimagnetic ordering, where cations within each sublattice are aligned parallel to one another, while antiparallel alignment exists between the A- and B-sublattices. Since the number of B-sites is twice that of A-sites, this magnetic arrangement results in a net magnetic moment, giving rise to ferrimagnetism. The magnetic properties of spinel ferrites can be effectively tuned by modifying the type of metal cations and their distribution between the A and B sites [38,39].

The aim of this review article is to provide a systematic analysis of the main synthesis methods for spinel-structured ferrite nanoparticles, their scientific principles, and the influence of synthesis parameters on the morphological, structural, and functional properties of the materials. The scope of the article covers chemical, physicochemical, and high-efficiency synthesis approaches for ferrite nanoparticles, along with their advantages and limitations, as well as the magnetic, electrical, optical, and resistive properties of the resulting nanostructures.

## 2. Synthesis Methods of Spinel Ferrite Nanoparticles

The synthesis of spinel ferrite nanoparticles involves a variety of techniques, including co-precipitation, sol–gel, hydrothermal methods, ball milling, wet chemical routes, thermal decomposition, ceramic methods, and solid-state synthesis. Accordingly, numerous synthesis strategies have been developed for the preparation of cobalt ferrite nanoparticles; however, no universal method exists for their synthesis. Each synthesis technique possesses its own advantages and limitations.

Two general approaches are employed for nanoparticle synthesis and nanostructure fabrication. The first is the top-down approach, in which bulk materials are broken down into nanoscale structures or particles by removing material from larger objects. This approach typically involves mechanical processes such as milling or attrition. The second is the bottom-up approach, where chemical or physical forces acting at the nanoscale are used to assemble basic building units into larger structures. A wide range of bottom-up techniques—such as hydrothermal and combustion synthesis—have been developed for the preparation of metal oxide nanomaterials.

**Figure 3:**
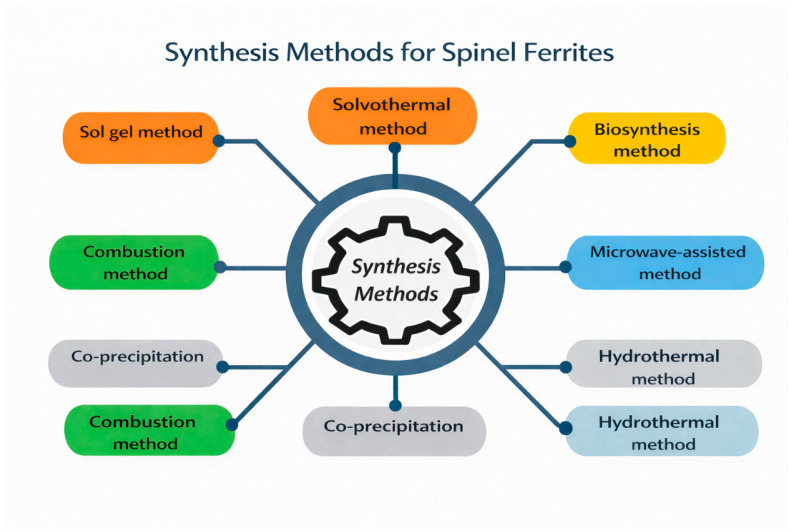
Schematic overview of chemical synthesis methods employed for nanoferrite preparation.

The main synthesis routes for spinel ferrites (MFe_2_O_4_) are illustrated (Figure 3), including the sol–gel, solvothermal, hydrothermal, co-precipitation, combustion, microwave-assisted, and biosynthesis methods. These approaches enable the controlled tuning of particle size, phase purity, and magnetic properties to meet specific application requirements.

### 2.1. Co-Precipitation Method

The co-precipitation method is a widely used technique for nanoparticle synthesis, in which precursors are dissolved in an aqueous solution followed by the addition of a base under continuous stirring in a reducing environment at room temperature, leading to the formation of a precipitate and subsequent nanoparticle formation. Process variables such as the type and ratio of salts used, pH, reaction temperature, and ionic strength allow control over the colloidal properties of the final particles [40]. Co-precipitation is a simple wet-chemical method that is extensively employed for the synthesis of spinel ferrites due to its low cost, simplicity, ability to produce fine particles, and good stoichiometric control. In this approach, metal salts are dissolved in common solvents in stoichiometric proportions, and a precipitating agent is added to achieve the desired pH, resulting in a single-phase homogeneous inorganic solid [41]. Aqueous solutions containing mixtures of divalent and trivalent transition metal ions are typically prepared in a 1:2 molar ratio [42,43]. Subsequently, sodium hydroxide is added under continuous stirring until the pH reaches 12, followed by the addition of oleic acid as a surfactant [44]. The resulting mixture is heated at 80 °C for 1 h and then cooled. The precipitate is washed and centrifuged to remove impurities, dried, and finally calcined at 600 °C for 10 h to obtain the desired nanoparticles [45]. Researchers have emphasized the use of high-purity chemicals and careful purification procedures to ensure particle purity. In one approach, Ristić and co-workers combined co-precipitation with autoclaving to produce nanoparticles, a process referred to as “combined synthesis” [46].

### 2.2. Combustion Method

The combustion method is one of the most efficient techniques for synthesizing a wide range of inorganic materials on an industrial scale [47]. Due to its high efficiency, straightforward implementation, low cost, and energy-saving nature, the combustion method has been widely adopted and intensively investigated within the scientific community. In this approach, spinel ferrite nanoparticles are produced through a highly exothermic oxidation–reduction (redox) reaction: metal nitrates (acting as oxidizers, including Fe(NO_3_)_3_·9H_2_O) are typically mixed with a fuel in stoichiometric proportions, and after gelation/thickening the mixture undergoes self-ignition, yielding a voluminous and porous (“fluffy”) powder. Although glycine is most commonly used as the fuel, citric acid, urea, and mixed fuel systems such as urea–glucose can also serve as effective alternatives [48]. Salunke et al. observed that this process is extremely rapid, with ignition and foam formation occurring within approximately 5 s [49]. By adjusting the glycine-to-nitrate ratio, parameters such as crystallite size and surface area can be controlled. The resulting product is typically voluminous and porous. In contrast, Prabhakaran and co-workers employed DL-alanine gel as a fuel in the combustion synthesis route [50]. Using this technique, DL-alanine was combined with metal and iron nitrate solutions to start a self-sustaining exothermic reaction. Metal ferrite was created by igniting the gel, and it was then calcined at various temperatures. Because it allows for the adjustment of factors that directly affect the final product, the combustion method provides a flexible approach for the synthesis of nanoparticles.

### 2.3. Sol–Gel Method

The sol–gel method is a chemical process in which solid particles are synthesized from colloidal suspensions through hydrolysis and polymerization reactions, involving the transformation of a liquid into a gel, followed by drying and heat treatment to obtain a dense material. This method provides high purity and homogeneity, operates at relatively low processing temperatures, and offers multifunctional material design with precise control over nanostructure [51]. In order to create spinel ferrite nanoparticles, metal alkoxide solutions are hydrolyzed and then condensation polymerized to create a gel. Heating is used to eliminate volatile components after synthesis. The creation of nanosized, homogenous, and highly active powders is made possible by the method’s many benefits, which include the use of cheap precursors, low energy consumption, ease of preparation, and minimal equipment requirements. However, careful control of reaction parameters such as calcination temperature, stirring rate, and solution concentration is required [42]. A major drawback is the potential impurity of the final product, which necessitates post-synthesis thermal treatment to achieve high purity. Nevertheless, the sol–gel method allows effective control over particle composition, size, homogeneity, and distribution [52].

### 2.4. Thermal Decomposition Method

Thermal decomposition refers to the process of breaking chemical bonds in compounds through the application of heat. This method is particularly useful for synthesizing monodisperse nanoparticles [53]. One of its main advantages is the provision of sufficient thermal energy to heal defects through high-temperature decomposition. Thermal decomposition techniques have been employed to control nanoparticle size, polydispersity, and morphology. However, these reactions are typically conducted under oxygen-free conditions at high temperatures using organic solvents and vapor phases to address safety concerns. Nanoparticles produced by oxygen-free thermal decomposition often exhibit inferior magnetic characteristics due to a broader distribution of magnetic diameters relative to their physical diameters [54].

### 2.5. Hydrothermal Method

In the hydrothermal synthesis method, soluble salts containing divalent and trivalent transition metals are dissolved separately and then combined in a 1:2 molar ratio [42]. To create a homogenous mixture, organic solvents like ethanol or ethylene glycol are progressively added to the aqueous solutions while being constantly stirred. After that, the mixture is put in an autoclave and cooked under intense pressure. The hydrothermal method seems to be one of the most promising methods for producing ferrite nanoparticles on a large scale when compared to other synthesis processes. By selecting appropriate solvent mixtures and varying temperature, pressure, and reaction time, nanoferrites with well-defined sizes and morphologies can be obtained [55,56].

### 2.6. Ceramic Method

The ceramic method is a simple and effective approach for synthesizing ferrite nanoparticles at very high temperatures. It involves grinding pre-certified oxides in an agate mortar, followed by pre-calcination at elevated temperatures to obtain the desired samples. Spinel ferrite powders prepared using this method are typically calcined at 1000 °C for up to 72 h [42].

### 2.7. Solid-State Reaction Method

The solid-state reaction method is an efficient and straightforward technique for nanoparticle synthesis, in which atoms or ions react through diffusion at high temperatures (typically above 800 °C). This method is suitable for large-scale production and can yield high-purity spinel ferrite nanoparticles; however, the reaction time is generally longer compared to other synthesis routes. It has been widely used to produce various types of nanoparticles [57].

### 2.8. Microwave

Microwave-assisted synthesis is another method used for the preparation of ferrite nanoparticles. In this technique, energy is directly delivered to materials through electromagnetic radiation, which is converted into thermal energy via molecular interactions, resulting in rapid heating. Typically, reaction temperatures range from 100 °C to 200 °C, and reaction times are significantly shorter than those of conventional methods. An exhaust system is employed to remove vapors generated during heating. Although microwave-assisted synthesis enables large-scale preparation of ferrite nanoparticles, its productivity is generally lower than that of thermal or hydrothermal decomposition and co-precipitation methods. Careful selection of reagents and precursors is essential to meet requirements related to cost-effectiveness and scalability [58].

### 2.9. Solvothermal Method

The solvothermal process has been widely employed due to its ability to precisely control particle size and morphology. Solvothermal synthesis refers to a chemical process carried out in non-aqueous solvents under elevated temperature and pressure conditions. Accordingly, XFe_2_O_4_ (X = Mn, Co, Ni) spinel ferrites were prepared via a solvothermal route in an ethylene glycol medium: stoichiometric amounts of analytical-grade metal chlorides (XCl_2_·nH_2_O) and FeCl_3_·6H_2_O were dissolved, and upon the addition of NaOH to induce hydroxide precipitation, the resulting mixture was treated in a Teflon-lined autoclave at 200 °C for 24 h. Finally, the products were purified by centrifugation, washed three times with ethanol and DI water, and dried under vacuum at 60 °C [59]. In this method, precursor solutions containing metal and iron salts are sealed in a high-pressure vessel and heated at high temperatures. The process yields nanoparticles with precise crystalline phases and controlled shape and size distributions. Parameters such as reaction time, temperature, surfactant type, solvent, and precursor composition can be adjusted. The simplicity of this method makes it particularly useful for preparing spinel ferrites with enhanced chemical and physical properties for biomedical and industrial applications [43].

### 2.10. Sonochemical Method

The sonochemical method is widely applied for the synthesis of spinel ferrite nanoparticles due to its ability to provide uniform mixing and easy control over reaction conditions. Ultrasonic waves, temperature, and intensity directly influence particle size, enabling the formation of crystalline phases at relatively low temperatures [60,61]. Ultrasonic-assisted sonochemical synthesis has emerged as a powerful, environmentally benign, and cost-effective alternative for nanoparticle fabrication. Compared with conventional approaches, this technique offers several key advantages, including accelerated reaction kinetics, enhanced product purity, and a narrower nanoparticle size distribution. The application of ultrasound can markedly reduce the final particle size and suppress agglomeration, thereby yielding more uniform products with improved crystallinity. Moreover, relative to high-temperature solid-state routes, sonochemical processing enables the formation of crystalline phases at substantially lower temperatures [62,63]. The sonochemical approach is of particular importance because it enables the production of high-purity materials with a narrow particle size distribution, offers an environmentally benign route, allows precise control over reaction conditions, and accelerates reaction kinetics, thereby facilitating the formation of nanoparticles with uniform morphologies. In addition, this method can enhance the performance of phase-transfer catalysts and improve their catalytic reactivity [64,65]. Notably, no calcination step is required in this process [66]. As a result, sonochemical synthesis offers advantages such as reduced particle size, improved uniformity, and high stability, although it also has certain drawbacks, including the need for specialized equipment and complex reaction conditions [67].

### 2.11. Electrochemical Method

The electrochemical synthesis of nanoparticles is similar to the co-precipitation method but utilizes ion sources generated through anodic dissolution of electrodes. This approach offers high-purity products and precise control over particle size. Sample purity and particle dimensions can be optimized by adjusting parameters such as electrode spacing and current density. Factors including pH, current density, electrolyte concentration, and electrode selection play crucial roles in achieving the desired quality of ferrite nanoparticles [68].

### 2.12. Mechanical Milling Method

In materials science, metallurgy, and nanotechnology, mechanical milling is a common solid-state powder processing method for creating alloyed materials and fine powders. In high-energy ball mills, powder particles are repeatedly fractured and cold-welded. This method is commonly employed to synthesize nanoferrites with a broad size distribution, typically ranging from 35 nm to 85 nm [69,70,71,72].

Table 1 summarizes and compares the most widely used synthesis routes for spinel ferrite nanoparticles in terms of processing temperature, typical particle size range, and the key advantages and limitations of each method. Overall, low-temperature wet-chemical approaches (e.g., co-precipitation, sol–gel, hydro/solvothermal, microwave-assisted) generally yield smaller particles with improved control, whereas high-temperature solid-state/ceramic routes tend to produce larger grains with limited tunability despite their scalability.

## 3. Characterization Techniques for Spinel Ferrite Nanoparticles

### 3.1. X-Ray Diffraction (XRD) Analysis

X-ray diffraction (XRD) is one of the most widely used techniques for the analysis of nanoparticles. Important information such as crystal structure, phase composition, lattice parameters, and crystallite size can be obtained from XRD measurements [73]. Using XRD analysis, the synthesized spinel ferrites are comparatively investigated in terms of crystal symmetry, unit cell parameters, pycnometric density, and X-ray density [74].

The intensities of diffraction peaks are calculated on a 100-point scale along the probability line. Data processing and calculations were performed using the PDXL/2 software [4]. For example, high calcination temperatures contribute to the elimination of structural defects or residual impurities, thereby improving crystal quality and promoting the growth of larger crystallites. The average crystallite size was estimated from the broadening of XRD peaks using the Debye–Scherrer equation, which is commonly applied for crystallite size determination [75]:

(1) $$D=\;\frac{k\lambda }{\beta cos\;\theta }$$

The microstructural characteristics of the synthesized complex ferrites, including crystallite size and microstrain, were further determined using the Williamson–Hall (W–H) method. The Williamson–Hall approach is a widely employed analytical technique for investigating crystalline structures and their mechanical properties. Based on XRD data, this method enables the evaluation of microstructural parameters, particularly the average crystallite size and lattice strain.

The Williamson–Hall equation is expressed as:

(2) $$\beta cos\;\theta =\;\frac{k\lambda }{D}+\;4\varepsilon sin\;\theta$$

where

β—Full width of the diffraction peak (also called “width”) in radians;
θ—Diffraction angle;
k—A system factor required for the calculation (usually taken as 0.9);
λ—Wavelength of the X-ray radiation;
D—Average crystallite size (sometimes referred to as “crystal size”);
ϵ—Effect of internal strain, i.e., the expansion and compression due to stresses within the crystal.

The W–H method generally yielded slightly larger crystallite sizes compared to those obtained using the Scherrer equation. This difference is mainly attributed to lattice strain effects induced by variations in calcination temperature.

**Figure 4:**
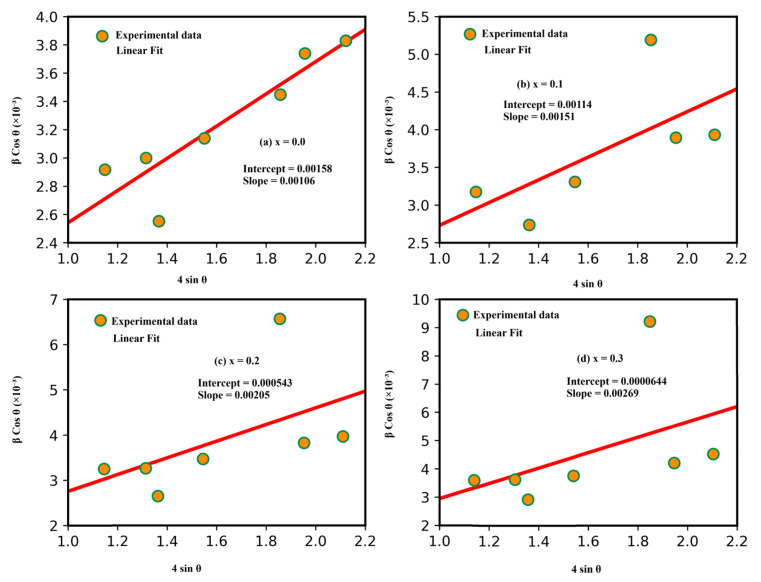
W–H plots of Cd_x_Li_0_._4−x_/_2_Ni_0_._2_Fe_2_._4−x_/_2_O_4_ samples at different Cd contents [76].

Figure 4 Williamson–Hall plots (βcos θ vs. 4 sin θ) and the corresponding linear fits are presented for different compositions (x = 0.0–0.3). The slope reflects the microstrain in the crystal lattice, while the intercept is used to estimate the crystallite size.

### 3.2. Density Determination of Spinel Ferrites

The experimental densities of the synthesized spinel ferrites were determined using the pycnometric method. Toluene was selected as the pycnometric liquid because it is chemically inert with respect to the investigated compounds and its density exhibits minimal variation with temperature. A pycnometer with a volume of 1 mL and an analytical balance with a precision of 0.0001 g were used. The experimental procedure was carried out as follows [74]:

- the empty pycnometer was weighed (M_0_);
- the pycnometer filled with distilled water was weighed (M_1_);
- the pycnometer filled with the pycnometric liquid (toluene) was weighed (M_2_);
- the pycnometer containing the sample was weighed (M_3_);
- the pycnometer containing the sample and filled with the pycnometric liquid was weighed (M_4_).

The density of the sample was then calculated using the following equation:

(3) $$\rho =\frac{M_{3}-M_{0}}{\frac{M_{1}-M_{2}}{\rho_{1}}-\frac{M_{4}-M_{3}}{\rho_{2}}}$$

where p_1_ is the density of water at 20 °C (0.9983 g·cm^−3^) and p_2_ is the density of the pycnometric liquid.

The density of the pycnometric liquid was calculated using the equation:

(4) $$\rho_{2}=\frac{M_{2}-M_{0}}{M_{1}-M_{0}}\cdot \rho_{1}$$

The X-ray density was calculated using the standard formula based on the XRD data:

(5) $$\rho_{rent}=\frac{1.66\cdot M_{c}\cdot Z}{V_{tor}}$$

where Z is the number of formula units per unit cell, M_(C)_ (cell) is the volume of the unit cell, and M_(C)_ is the molecular weight of the compound.

### 3.3. Scanning Electron Microscopy (SEM)

Scanning electron microscopy (SEM) is one of the most important techniques used to investigate the morphology and surface structural features of nanosized ferrites. This method provides high-resolution images that allow determination of particle size and shape [77,78,79,80,81,82,83,84,85,86].

SEM analysis enables visualization of nanostructural features, particularly agglomerated structures, which is crucial for understanding the synthesis process and its influence on the final material properties [87,88,89]. In addition, SEM offers valuable information on the porosity of ferrite nanostructures. Porosity plays a significant role in applications such as catalysis and magnetic devices, as it directly affects the functional performance of the material [87,90].

**Figure 5:**
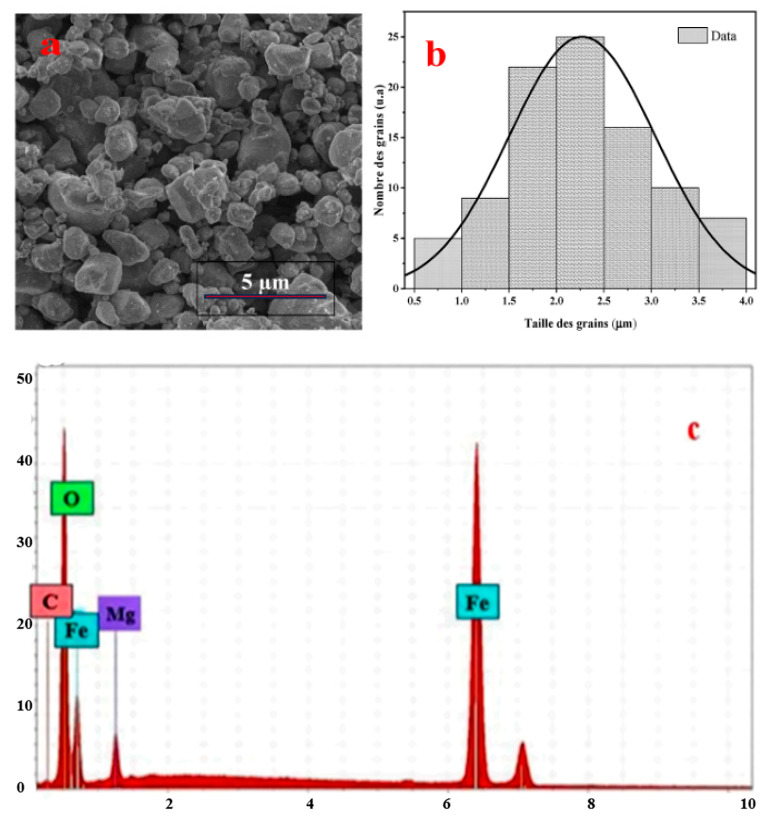
SEM image (**a**), particle size distribution (**b**), and EDX spectra (**c**) of LiMg_0.5_Fe_2_O_4_.

By combining SEM with energy-dispersive X-ray spectroscopy (EDS), the elemental composition of ferrites can be examined, confirming the presence of the desired elements and their distribution within the nanostructure [87,91]. The average particle sizes of the ferrite nanoparticles were estimated, and the obtained results are consistent with those derived from XRD analysis [87]. In Figure 5a, the LiMg_0.5_Fe_2_O_4_ spinel ferrite particles appear as irregularly shaped grain agglomerates with heterogeneous grain sizes, indicating a rough and microporous surface morphology. The agglomeration is attributed to the large specific surface area of the particles and their interaction through weak van der Waals forces. The histogram in Figure 5b shows an average grain size of approximately 2.27 μm, confirming that the solid-state synthesis produces micrometer-sized grains. The energy-dispersive spectrum (Figure 5c) and the quantitative data in Table 2 confirm the presence of the required Mg, Fe, and O elements and demonstrate good agreement between the experimental composition and the theoretical values [92].

The absence of the Li-related peak is attributed to the detection limit of the instrument. The carbon-related peak originates from the sample substrate. The absence of foreign elements confirms the successful synthesis of the LiMg_0.5_Fe_2_O_4_ material. Furthermore, Li is isomorphically substituted for Mg; thus, lithium atoms occupy the Mg lattice sites.

### 3.4. FTIR Analysis

Fourier transform infrared (FTIR) spectroscopy is employed to identify functional groups and metal–oxygen chemical bonds in nanosized ferrites and plays a crucial role in confirming the formation of the spinel structure [93]. Typically, absorption bands observed in the FTIR spectra around ~600 cm^−1^ and ~425 cm^−1^ correspond to the stretching vibrations of metal–oxygen bonds at tetrahedral and octahedral sites, respectively [94]. These characteristic bands indicate the successful formation of the spinel ferrite phase.

**Figure 6:**
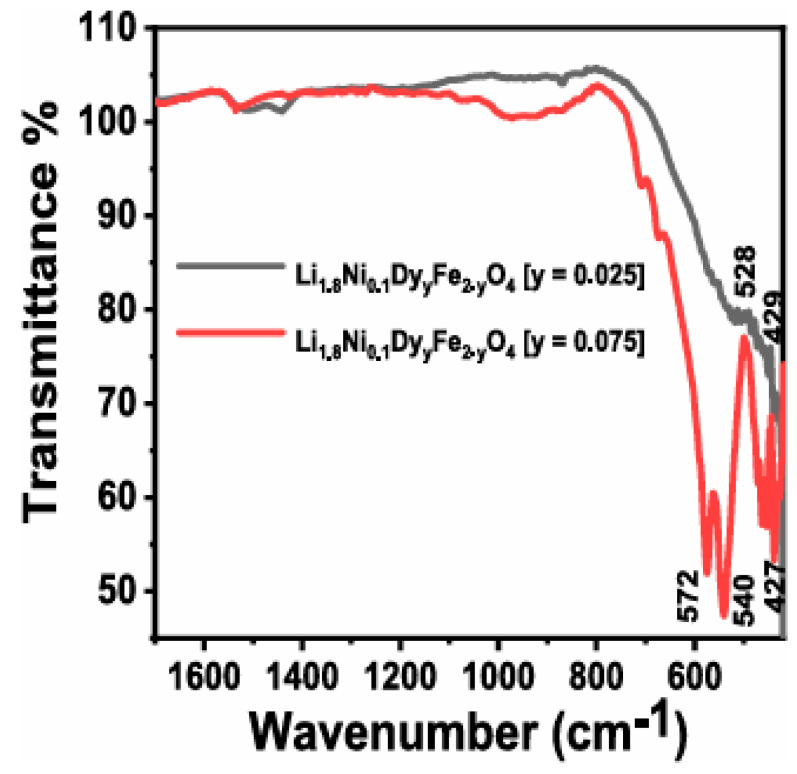
FTIR spectra of Li_1_._8_Ni_0_._1_Dy_γ_Fe_2−__γ_O_4_ (y = 0.025, 0.075) compounds.

In addition, shifts in the absorption bands reflect changes in the local environment of metal ions, which can influence the magnetic and dielectric properties of ferrites [93]. FTIR analysis enables the assessment of the purity of synthesized nanoferrites and facilitates investigation of the effects of dopant elements on structural properties [95,96]. For instance, as shown in Figure 6, the FTIR spectra of Li_1_._8_Ni_0_._1_Dy_γ_Fe_2−__γ_O_4_ (y = 0.025 and 0.075) compounds confirm the characteristic features of a spinel ferrite structure. The absorption bands in the 600–400 cm^−1^ region are attributed to Fe–O bond vibrations, while an increase in Dy content leads to slight band shifts, indicating structural modifications within the crystal lattice [97].

### 3.5. FT-Raman

Raman spectroscopy is considered an effective analytical technique for investigating the vibrational modes within the crystal structure of ferrite materials. This method enables confirmation of the formation of the spinel phase during the synthesis process and plays a crucial role in evaluating the structural integrity and functional properties of the material. Since the FT-Raman technique allows the analysis of nanoscale ferrites without altering their physical or chemical state, it is particularly well suited for the characterization of delicate nanostructures [87].

In addition, this technique provides valuable information on internal stresses and structural defects in the synthesized ferrites. As these factors significantly influence the magnetic and electronic properties of the materials, their identification is essential for optimizing synthesis conditions. FT-Raman spectroscopy also makes it possible to examine the effect of dopant elements on the vibrational characteristics of ferrites, thereby offering insight into how different substituting ions modify the material properties and overall performance.

**Figure 7:**
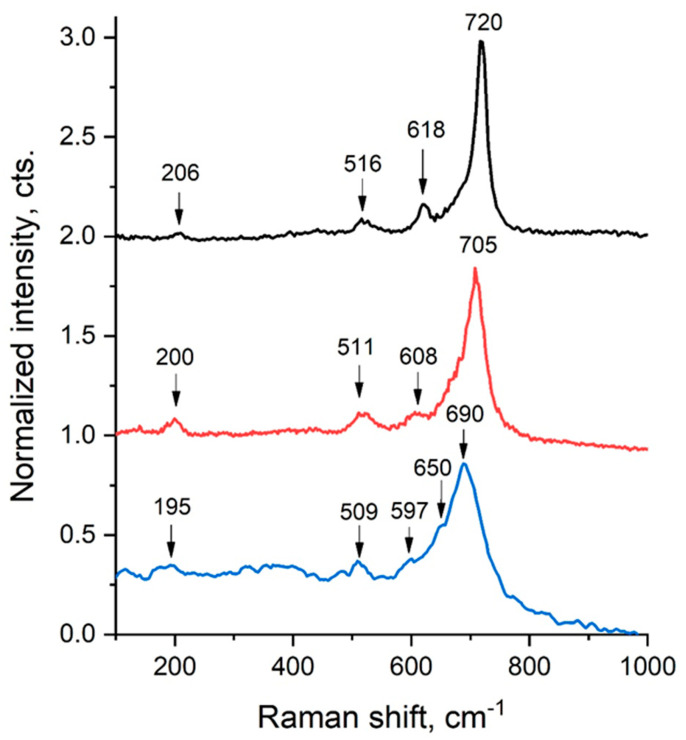
Raman Spectrum of the LiCr_3_._4_Fe_1_._6_O_8_ Sample.

As an example, Figure 7 presents three Raman spectra. The peaks observed near 200 cm^−1^ (195, 200, and 206 cm^−1^) are attributed to lattice vibrations involving Li^+^, Cr^3+^, or Fe^3+^ cations. The bands appearing in the 500–600 cm^−1^ region (509, 511, 516, and 597 cm^−1^) correspond to Fe–O stretching vibrations in the spinel lattice. The peaks identified in the 600–700 cm^−1^ range (608, 618, 650, 690, and 705 cm^−1^) are associated with Fe–O vibrational modes in ferrite groups, particularly those related to CrO_4_^2−^ units. A pronounced band around 720 cm^−1^ indicates a high concentration of ferrite groups [98]. Furthermore, FT-Raman spectroscopy can be applied to investigate the temperature dependence of vibrational modes, allowing the thermal stability and possible phase transitions of nanoscale ferrites to be determined. By analyzing the position and intensity of Raman bands, this technique serves as a reliable tool for quality control of synthesized nanoferrites, ensuring their suitability for various practical applications.

### 3.6. Dielectric Properties

The dielectric properties of nanosized ferrites represent one of the key factors determining their effectiveness in electronic device applications, as these parameters directly influence energy storage capability and signal transmission quality [87,99]. The dielectric constant (ε) and dielectric loss tangent (tan δ) are regarded as the principal indicators describing the insulating behavior of ferrite materials, and they are of particular importance for applications in electronic components such as capacitors and inductive elements [99,100].

**Figure 8:**
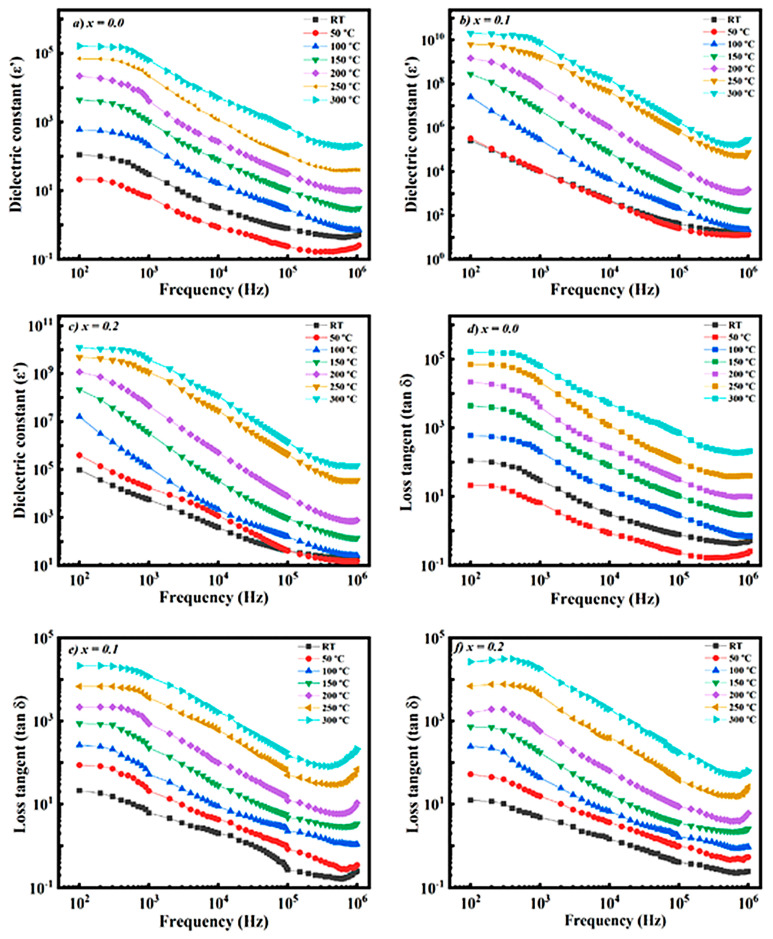
Frequency dependence of the dielectric constant (**a**–**c**) and dielectric loss factor (tan δ) (**d**–**f**) at selected temperatures.

Investigation of dielectric characteristics makes it possible to evaluate the influence of microstructural features of ferrites, including grain size, porosity level, and overall electrical behavior [100,101]. Moreover, analysis of the variation in dielectric parameters over a wide frequency range provides deeper insight into the electrical response of nanoferrites intended for high-frequency device applications [99]. For this purpose, the frequency- and temperature-dependent behavior of the dielectric constant (ε′) was examined, and the obtained results are presented in graphical form. For instance, the results derived from Figure 8 indicate that both the dielectric constant (ε′) and the dielectric loss factor (tan δ) decrease with increasing frequency, whereas an increase in temperature and Cd-ion concentration enhances charge carrier activity, leading to more pronounced polarization and relaxation mechanisms. These observations confirm that the investigated materials are promising candidates for high-temperature and high-frequency electronic applications [76].

**Figure 9:**
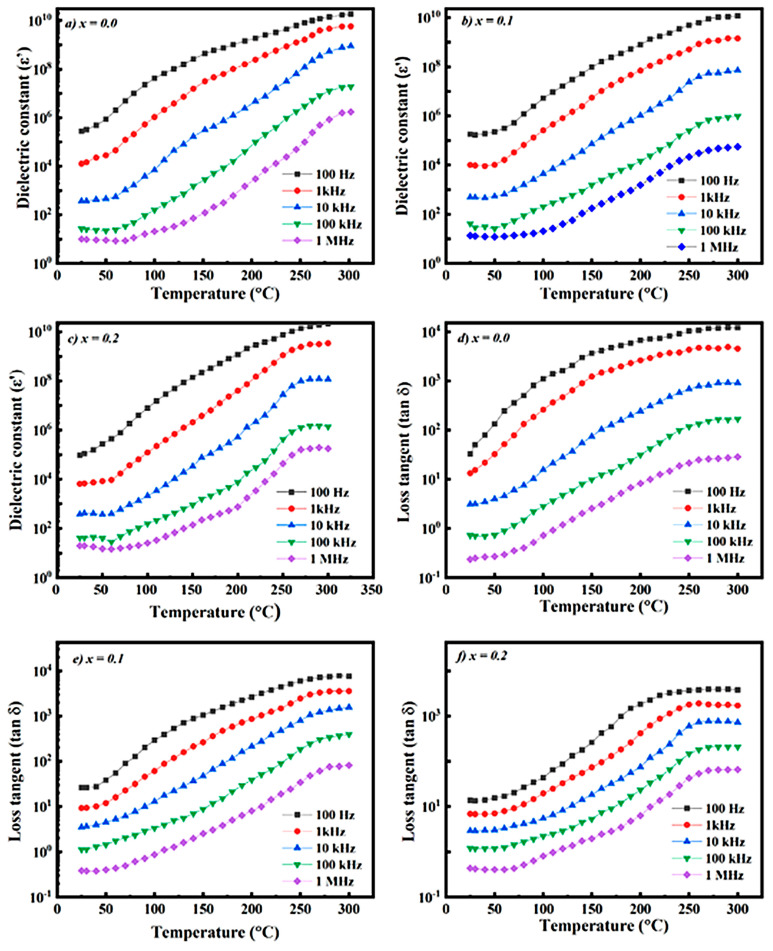
Temperature dependence of the dielectric constant (**a**–**c**) and dielectric loss tangent tan δ (**d**–**f**) at selected frequencies.

As illustrated in Figure 9, the dielectric constant (ε′) of all samples increases with increasing temperature, while it decreases with increasing frequency. This behavior indicates the dominance of space charge and interfacial polarization mechanisms at low frequencies. The dielectric loss tangent (tan δ) also exhibits a temperature-dependent increase, whereas it shows significantly lower values at higher frequencies. An increase in Cr concentration leads to a reduction in both ε′ and tan δ values, suggesting an improvement in the electrical stability of the material. These results demonstrate that the investigated materials are suitable candidates for high-frequency and temperature-resistant electronic applications [76].

### 3.7. Magnetic Properties

The magnetic properties of nanosized ferrites are commonly investigated using advanced measurement techniques such as vibrating sample magnetometry (VSM) and superconducting quantum interference device (SQUID) magnetometry. These methods enable accurate determination of key magnetic parameters, including saturation magnetization, coercivity, and remanent magnetization, which play a decisive role in describing the magnetic behavior of materials [101].

The hysteresis loops obtained from magnetic measurements provide essential information on the magnetic nature of nanoferrites. The shape and area of the hysteresis loop reflect the coercivity and remanent magnetization levels of the material, parameters that are of particular importance for applications in magnetic memory devices, sensors, and other functional systems [102,103,104].

The magnetic properties of nanoferrites vary with temperature, including their dependence on the Curie temperature. Therefore, temperature-dependent magnetization is frequently examined to evaluate phase transitions and the thermal stability of the material, which is especially critical for devices operating under high-temperature conditions [89]. In this context, elucidating the correlation between structural parameters—such as crystallite size and morphology—and magnetic properties is of great interest. To this end, X-ray diffraction (XRD) and scanning electron microscopy (SEM) are often employed in conjunction with magnetic measurements to analyze the relationship between structural features and magnetic behavior [101]. Such comprehensive studies enable a deeper understanding of the magnetic properties of nanoferrites and facilitate their efficient utilization in electronics, magnetic recording systems, and biomedical applications [103].

**Figure 10:**
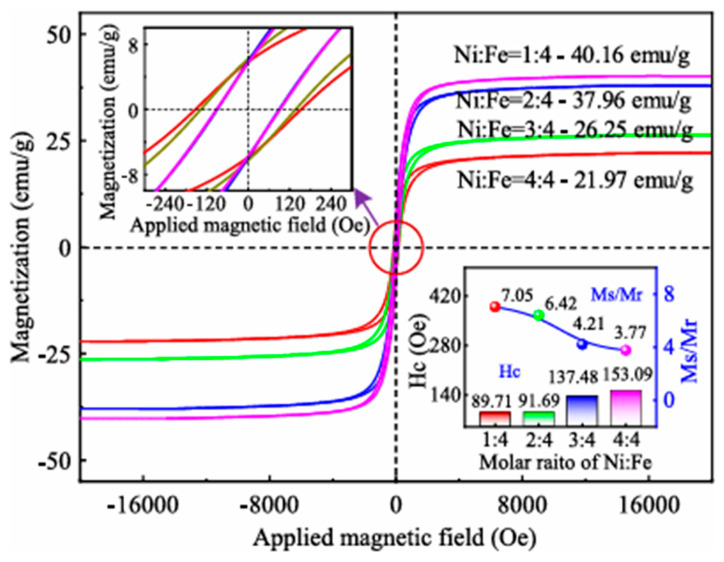
Magnetization hysteresis loops of synthetic Ni_x_Fe_3-x_O_4_ samples roasted at an oxidation roasting temperature range of 1200 °C for a roasting time of 2.0 h with different molar ratios of Ni/Fe in an air atmosphere.

Spinel ferrites (SFs) can be categorized into six main groups—paramagnetic, diamagnetic, ferrimagnetic, ferromagnetic, antiferromagnetic, and superparamagnetic—depending on the type (magnetic or non-magnetic) and distribution of cations at the tetrahedral (A) and octahedral (B) sites. For instance, Figure 10 shows the three main magnetic properties of spinel ferrites: coercive field (Hc), remanent magnetization (Mr), and saturation magnetization (Ms).

A high saturation magnetization (Ms) combined with negligible coercive field (Hc) is characteristic of superparamagnetic behavior, a property of particular importance for water treatment applications. Superparamagnetism is typically observed in small ferromagnetic or ferrimagnetic nanoparticles with diameters below approximately 3–50 nm, depending on the material [104]. For example, Figure 10 shows the magnetization hysteresis loops of synthetic Ni_x_Fe_3−x_O_4_ samples with different Ni/Fe molar ratios, roasted in an air atmosphere at 1200 °C for 2.0 h. As the Ni:Fe ratio increases from 1:4 to 2:4, the saturation magnetization (M_s_) gradually decreases from 40.16 to 37.96 emu/g, whereas for ratios higher than 2:4, M_s_ markedly drops to 21.97 emu/g. This behavior is attributed to the increased concentration of Ni^2+^ ions, which inhibits grain growth, leading to a reduction in grain size; additionally, the fraction of non-collinear magnetic structures in the surface layer increases, resulting in a lower saturation magnetization. Moreover, the coercivity (Hc) increases from 89.71 to 153.09 Oe as the Ni:Fe ratio rises from 1:4 to 4:4, while the M_s_/M_r_ ratio decreases from 7.05 to 3.77. The continuous increase in remanent magnetization suggests that the magnetic domain size (approximately equal to the grain size) gradually decreases and approaches the critical dimension for the transition to single-domain behavior [105].

### 3.8. Optical Characterization

Two main techniques are commonly employed to investigate the optical properties of spinel ferrites: ultraviolet–visible (UV–Vis) spectroscopy and photoluminescence (PL) spectroscopy. UV–Vis spectroscopy enables the determination of the light absorption characteristics of spinel ferrites in the wavelength range of 200–800 nm. Based on the obtained absorption data, the Tauc relation is applied to estimate the direct optical band gap energy of the material. According to the literature, the direct band gap energies of spinel ferrites determined using the Tauc method typically lie in the range of approximately 1.7–4.0 eV [39,106,107].

Photoluminescence spectroscopy is widely used to investigate structural defects in spinel ferrites, including lattice imperfections and oxygen vacancies. PL emission spectra recorded at a specific excitation wavelength are closely related to the defect states of the material. Several studies have reported characteristic PL emission bands of spinel ferrites in the range of about 450–530 nm, which are attributed to crystallographic defects and oxygen deficiency in the lattice [39,106,107].

## 4. Applications of Spinel Ferrites

**Figure 11:**
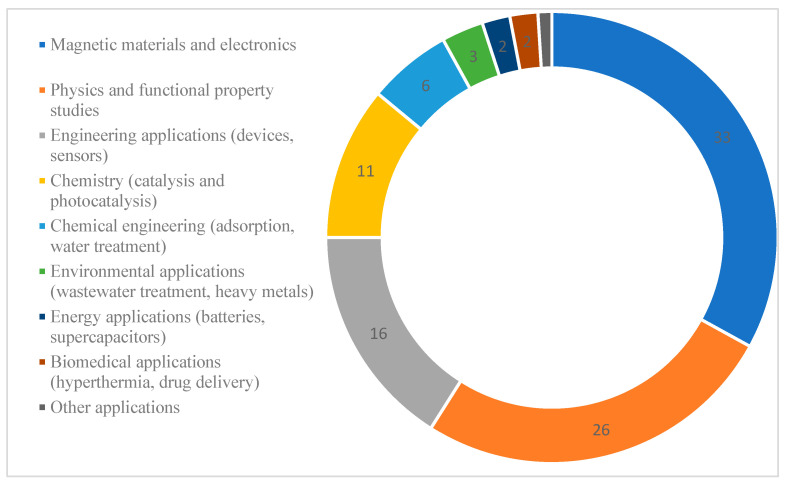
Quantitative comparison of application areas of spinel ferrites.

Spinel ferrites (SFs) are among the materials that exhibit broad applicability across various scientific and technological fields due to the synergistic combination of their structural, electrical, and magnetic properties. The diverse application areas of these materials are summarized schematically in Figure 11. In particular, spinel ferrites have been traditionally used as magnetic materials. In addition, they have been extensively investigated as efficient adsorbents for the removal of heavy metal ions and organic dyes in water and wastewater treatment, as well as catalysts and photocatalysts for the degradation of various pollutants.

Furthermore, spinel ferrites show high potential in biomedical applications, including their use as contrast agents, magnetic hyperthermia materials, and systems for drug delivery and controlled release. They are also suitable for use as sensors and biosensors in biotechnology and medical fields, as functional materials in electronics and microwave devices, in electromagnetic interference (EMI) shielding systems, and as magnetic recording media.

### 4.1. Microwave Devices

MnZn ferrites and zinc-substituted cobalt spinel ferrites are characterized by low dielectric losses, which makes them highly suitable for microwave device applications. Low losses reduce energy dissipation and enhance device efficiency. In addition, these materials exhibit stable performance over a wide frequency range and possess high mechanical strength and chemical stability, thereby extending the service lifetime of microwave devices [108,109].

### 4.2. Telecommunication Applications

Spinel ferrite nanoparticles, particularly Zn-doped CoFe_2_O_4_, are widely used in telecommunication systems due to their low power losses. They are effective in the fabrication of inductive components and transformers, improving signal filtering and amplification processes. Furthermore, the integration of ferrites into antenna structures broadens the operating bandwidth and enhances the overall performance of telecommunication systems [108,109].

### 4.3. Dielectric Applications

Spinel ferrites with high dielectric constants and low dielectric losses are effectively employed in capacitors and high-frequency electronic components. The combination of magnetic and dielectric properties makes them promising magnetodielectric materials for sensors and actuators. Moreover, these materials retain their dielectric characteristics over a wide temperature range, ensuring reliable performance under various operating conditions [109,110].

### 4.4. Water Treatment

ZnFe_2_O_4_ and CoFe_2_O_4_ spinel ferrites exhibit high efficiency in the photocatalytic degradation of organic dyes. Owing to their magnetic properties, these nanoparticles can be easily separated from water and reused. In addition, composites combined with silver nanoparticles or other functional materials enhance antibacterial and catalytic activity, enabling the development of environmentally friendly water treatment technologies [111,112,113].

### 4.5. Biomedical Applications

Spinel ferrites such as MnFe_2_O_4_ are effectively utilized in targeted drug delivery systems due to their superparamagnetic properties, allowing precise control of drug release under an external magnetic field. Furthermore, ferrite nanoparticles serve as efficient contrast agents for magnetic resonance imaging (MRI) and are considered promising materials for biosensors and antibacterial medical coatings [93,95,107,114].

Spinel-structured magnetic ferrite nanoparticles (CuFe_2_O_4_, MnFe_2_O_4_, CoFe_2_O_4_, MgFe_2_O_4_, ZnFe_2_O_4_, Fe_3_O_4_, NiFe_2_O_4_, and mixed ferrites) are extensively investigated as targeted drug-delivery platforms. Their tunable surface chemistry, (super)paramagnetic behavior, and the possibility of externally field-guided/targeted release broaden their biomedical applicability. Key functionalization approaches include polymeric coatings (e.g., PLA, PEG), bioconjugation with biomolecules (e.g., folate, doxorubicin), and hybridization with carbon nanomaterials; collectively, these strategies enhance colloidal stability, targeting capability, and multifunctionality of the system [115,116].

Specific examples include CuFe_2_O_4_ coated with poly(methacrylic acid) and lignin derivatives, which enables pH-dependent release of curcumin and exhibits pronounced cytotoxicity against breast cancer cells [117]. Folic-acid-modified MnFe_2_O_4_ systems provide sustained doxorubicin release and improve MRI contrast performance [118]. L-cysteine-functionalized CoFe_2_O_4_ demonstrates high drug-loading capacity and pH-responsive release, making it promising for theranostic applications; however, its relatively higher cytotoxicity requires further optimization [119]. MgFe_2_O_4_-based composites can effectively load antibiotics and show strong antibacterial activity via pH-triggered release [120,121]. ZnFe_2_O_4_ nanoparticles deliver curcumin efficiently and induce anticancer effects while maintaining low toxicity and high biocompatibility [122,123]. Fe_3_O_4_ coated with chitosan and gallic acid achieves pH-sensitive therapeutic release without compromising biocompatibility [124]. Hollow NiFe_2_O_4_ nanospheres and coated NiFe_2_O_4_ carriers exhibit high loading capacity and a combined therapy–imaging (therapeutic–diagnostic imaging) function, although surface modification is needed to mitigate potential nickel-associated toxicity [125]. In addition, systems such as CaFe_2_O_4_ and Zn-doped MgFe_2_O_4_ show controllable release profiles and antibacterial efficacy, supporting the potential of mixed ferrites as “tailor-made” nanocarriers [123,126]. Overall, ferrite nanomaterials optimized via green synthesis and surface engineering are regarded as safe and effective multifunctional candidates for drug delivery [115,116,117,118,119,120,121,122,123,124,125,126].

### 4.6. Gas Sensors

Spinel ferrite nanomaterials are widely applied in sensors for detecting gases such as H_2_S, NH_3_, and C_2_H_5_OH. Doping with metal ions significantly enhances their sensitivity and selectivity. An increase in operating temperature accelerates reaction kinetics and improves sensor response, while the magnetic properties enable easy recovery and reuse of the sensors [127].

### 4.7. Catalytic Applications

Spinel ferrite nanoparticles are extensively used in environmental remediation and organic synthesis processes due to their high catalytic activity and magnetic recoverability. Surface modification and composite structures can further enhance their adsorption and photocatalytic properties. Moreover, they are promising catalysts in photoelectrochemical systems such as water photolysis and hydrogen production [31,111,128].

### 4.8. Photoluminescent Applications

The photoluminescent properties of spinel ferrites can be tuned by varying their composition and particle size. Doping with rare-earth elements or transition metals enhances emission efficiency, enabling their use in display technologies, lighting devices, and bioimaging applications [32,93,108].

### 4.9. Data Storage Technologies

Core–shell structured spinel ferrite nanoparticles exhibit controllable coercivity, making them suitable for high-density magnetic recording systems. These characteristics render them promising materials for next-generation data storage devices [129].

### 4.10. Acid Mine Drainage (AMD) Treatment

Spinel ferrites are employed in the treatment of acid mine drainage through the efficient removal of heavy metals such as Zn, Cu, and As. This process results in the formation of chemically stable ferrite sludges, preventing the secondary release of toxic elements and ensuring environmental safety [130].

### 4.11. Voltage-Tunable Inductors

Ni–Cu–Zn spinel ferrites, when incorporated into magnetoelectric composites, enable voltage-controlled tuning of magnetic permeability. This significantly improves the tuning efficiency of inductors and offers high-performance solutions for modern microelectronic devices [131].

### 4.12. Magnetic Hyperthermia Using Spinel Ferrites

Magnetic hyperthermia aims to selectively damage tumor tissue by raising the local temperature to 40–45 °C using magnetic nanoparticles exposed to an alternating magnetic field (AMF). Heat is generated via Néel/Brownian relaxation and/or hysteresis losses. Surface functionalization improves tumor targeting, enhances treatment precision, and can reduce systemic side effects.

Among spinel ferrites, CuFe_2_O_4_ exhibits high saturation magnetization and may provide synergistic outcomes when combined with drug delivery or photothermal therapy. MnFe_2_O_4_ modified with PEG, folate, or thermoresponsive polymers enables controlled heating, temperature-triggered drug release, and enhanced MRI contrast, supporting multifunctional theranostic platforms. Owing to high magnetic anisotropy, CoFe_2_O_4_ can facilitate hyperthermia-triggered doxorubicin release and combined treatment strategies. SPIONs remain among the most clinically advanced candidates, offering efficient heating and chemo–hyperthermia synergy through tunable coatings. MgFe_2_O_4_ and ZnFe_2_O_4_ show favorable biocompatibility with effective heating, and their performance can be further improved by doping and surface engineering. NiFe_2_O_4_ can generate strong heating due to anisotropy, but requires biocompatible coatings to mitigate potential cytotoxicity. In addition, polymer- or silica-coated mixed ferrites can provide pH- and temperature-responsive drug release under AMF, enabling multimodal therapy with synergistic cytotoxicity in tumor cells. Overall, controlled synthesis and surface engineering are essential to design safe and efficient ferrite-based hyperthermia systems [132].

## 5. Advantages and Limitations

Spinel ferrites (SFs) have attracted considerable attention from the scientific community in recent years due to their unique combination of electrical, dielectric, electronic, mechanical, magnetic, optical, and catalytic properties [133]. As these materials simultaneously exhibit ferrimagnetic and semiconducting behavior, they can be classified as magnetic semiconductors [134]. Spinel ferrites are distinguished by exceptional physicochemical characteristics, including high Curie temperature, pronounced magnetocrystalline anisotropy, good thermal stability, and favorable chemical composition.

In addition, spinel ferrites possess excellent magnetic properties, controllable particle size and shape, high specific surface area, abundant active surface sites, high chemical stability, and ease of modification or functionalization [134]. These properties largely depend on the nature of the divalent cations (other than Fe^3+^) in the spinel structure, their distribution between tetrahedral and octahedral sites, as well as the synthesis method, grain size, morphology, and calcination temperature employed [135,136,137].

**Figure 12:**
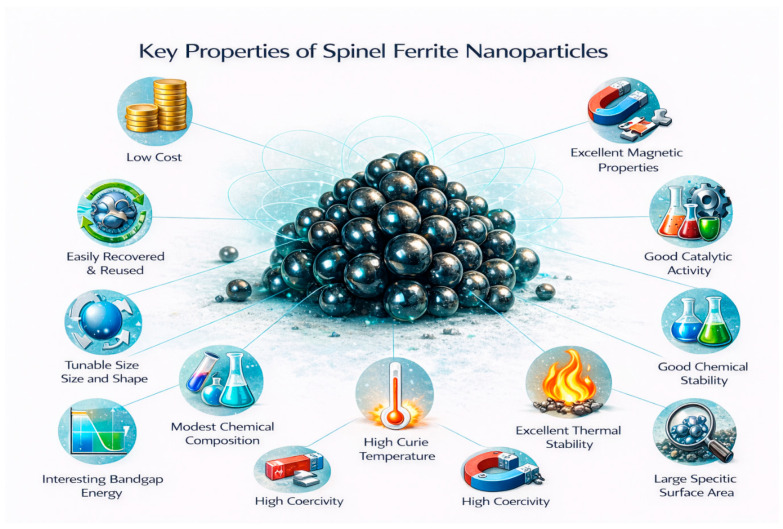
Various properties of spinel ferrite nanoparticles.

In recent years, spinel ferrites have been extensively investigated for water treatment and environmental protection applications owing to their low production cost, high catalytic activity, attractive band gap energies, good selectivity, recyclability and reusability without significant loss of activity, and relatively low toxicity [138]. Figure 12 schematically summarizes the main advantages of spinel ferrite nanoparticles.

## 6. Future Directions and Challenges

Future research on spinel-structured ferrite nanoparticles should focus on achieving a deeper understanding of the structure–property–application relationships and on the deliberate control of these correlations. In particular, precise regulation of cation distribution between the tetrahedral (A) and octahedral (B) sites represents a key strategy for tuning magnetic anisotropy, coercivity, dielectric stability, and optical response, thereby enabling the development of highly functional materials. Moreover, nanoscale control over particle size and morphology can optimize superparamagnetic behavior and open new opportunities for applications in high-frequency electronics, gas sensors, and biomedical systems.

In forthcoming studies, the development of environmentally friendly, energy-efficient, and industrially scalable synthesis routes will play a crucial role. The design of hybrid and composite systems—including core–shell architectures, surface-functionalized structures, and multicomponent ferrites—can significantly enhance catalytic, photocatalytic, and sensing performance. In addition, data-driven modeling and machine learning approaches are emerging as promising tools for predicting complex relationships between synthesis parameters and material properties.

Despite these prospects, several critical challenges remain for the practical implementation of spinel ferrite nanoparticles. These include their tendency toward agglomeration, broad particle size distributions, difficulties in ensuring synthesis reproducibility, and issues related to long-term chemical and thermal stability. For biomedical applications, comprehensive evaluation of toxicity, biocompatibility, and in vivo stability remains one of the major limiting factors.

## 7. Future Outlook/Challenges

Future direction: Establish robust structure–property–application relationships and precisely control cation distribution (A/B sites) as well as particle size and morphology to tailor ferrite performance.

Key challenges: Persistent agglomeration, broad size distribution, limited synthetic reproducibility, and insufficient long-term chemical/thermal stability.

Biomedical bottlenecks: Comprehensive evaluation of toxicity, biocompatibility, and in vivo stability, alongside optimization of safe surface coatings/functionalization, remains essential for translation.

## 8. Conclusions

This review systematically examined the synthesis routes, crystalline-structure characteristics, and structure–property–application relationships of spinel-structured ferrite nanoparticles. Various chemical and physicochemical approaches were compared in terms of their impact on particle size, morphology, cation distribution over A/B sites, and the degree of inversion, highlighting that these factors play a decisive role in determining magnetic, dielectric, optical, and catalytic properties.

The analysis indicates that the functional performance of spinel ferrites is governed not only by chemical composition but also by synthesis-dependent parameters such as crystal defects, oxygen vacancies, and surface state. Therefore, achieving application-specific properties requires precise control of cation distribution and nanoparticle size, mitigation of agglomeration, and improved structural homogeneity.

Despite the strong potential of spinel ferrite nanoparticles, several practical limitations remain. These include a tendency toward agglomeration, broad particle-size distributions, insufficient synthetic reproducibility, and challenges related to long-term chemical/thermal stability. For biomedical applications in particular, comprehensive assessment of toxicity, biocompatibility, and in vivo stability is essential and remains a key requirement for translation.

Overall, spinel ferrites can evolve into high-performance functional materials across diverse fields when synthesis is tightly controlled and surface engineering is effectively implemented. Future studies should strengthen property-by-design strategies by clarifying the links between cation distribution–defects–surface state–properties, adopting standardized characterization and measurement protocols, and addressing safety considerations more rigorously to accelerate reliable deployment of spinel ferrites in real-world applications.

## Figures and Tables

**Table 1:** Comparison of synthesis methods, processing temperatures, particle sizes, advantages, and disadvantages for spinel ferrite nanoparticles.

Synthesis Method	Temperature (°C)	Particle Size (nm)	Advantages	Disadvantages
Co-precipitation	Room temp–100	5–50	Low cost, simple process, good stoichiometric control, scalable	Agglomeration, limited size control
Combustion	300–800	10–60	Fast synthesis, energy efficient, low cost	Porous structure, poor morphology control
Sol–gel	200–600	5–40	High purity, homogeneous particles, good size control	Requires calcination, sensitive parameters
Thermal Decomposition	200–350	5–20	Monodisperse particles, excellent size control	High temperature, toxic solvents
Hydrothermal	120–250	10–80	High crystallinity, controlled morphology	Long reaction time, autoclave required
Ceramic Method	900–1200	>100	Simple, suitable for bulk production	Large particle size, poor control
Solid-State Reaction	800–1200	>100	High purity, industrially scalable	Long processing time, coarse particles
Microwave-assisted	100–200	10–50	Short reaction time, energy efficient	Limited scalability, lower yield
Solvothermal	150–300	10–50	Good size and shape control, high crystallinity	Expensive solvents, high pressure
Sonochemical	<100	5–30	Low temperature, uniform particles, eco-friendly	Specialized equipment required
Electrochemical	Room temp–80	10–40	High purity, controlled particle size	Sensitive to pH and current density

**Table 2:** Atomic masses, percentages, and the corresponding theoretical values of LiMg_0_._5_Fe_2_O_4_.

Element	Experimental Mass (%)	Experimental Atomic (%)	Theoretical Mass (%)	Theoretical Atomic (%)
Carbon	1.35	5.26	–	–
Oxygen	33.15	57.22	32.96	53.33
Magnesium	4.59	6.30	6.25	6.66
Iron	57.91	31.22	57.51	26.66

## Data Availability

The original contributions presented in the study are included in the article; further inquiries can be directed to the corresponding author.
